# ﻿Underestimated cryptic diversity in the *Caryocolumtricolorella* species complex (Lepidoptera, Gelechiidae)

**DOI:** 10.3897/zookeys.1103.83952

**Published:** 2022-06-03

**Authors:** Peter Huemer

**Affiliations:** 1 Tiroler Landesmuseen Betriebgsges.m.b.H., Sammlungs- und Forschungszentrum, Naturwissenschaftliche Sammlungen, Krajnc-Str. 1, A-6060 Hall in Tirol, Innsbruck, Austria Tiroler Landesmuseen Betriebgsges.m.b.H., Sammlungs- und Forschungszentrum Innsbruck Austria

**Keywords:** DNA barcode, Europe, Gelechiinae, morphology, new species, vicariant distribution

## Abstract

The taxonomy of the *Caryocolumtricolorella* species complex, an informal subsection of the diverse *Caryocoluminteralbicella* species group, is revised and four species are separated from DNA barcodes of the mitochondrial COI (cytochrome c oxidase subunit 1) gene and adult morphology: *C.tricolorella* (Haworth, 1812), *C.fibigerium* Huemer, 1988, *C.herwigvanstaai***sp. nov.**, and *C.olekarsholti***sp. nov.** These species show a vicariant distribution pattern, with *C.tricolorella* widely distributed in Central and Northern Europe, *C.fibigerium* restricted to the Iberian Peninsula and southern France, *C.herwigvanstaai***sp. nov.** to the Italian Peninsula, and *C.olekarsholti***sp. nov.** to the Balkans. All species are described in detail, and the adults and genitalia of both sexes are illustrated.

## ﻿Introduction

The European fauna of Lepidoptera is generally considered as well explored, although about 50 species are still described as new to science yearly (www.lepiform.de). However, the species diversity of some families of so-called microlepidoptera seems insufficiently documented. An extraordinarily high portion of potentially overlooked cryptic diversity is found, for example, in the Gracillariidae and Gelechiidae, with an estimated proportion of up to 10% of undescribed species for both families (Huemer et al. 2020; Lopez-Vaamonde et al. 2021).

With currently about 870 described species, the Gelechiidae are among the most diverse families of Lepidoptera in Europe (Huemer and Karsholt 2020), but despite considerable progress in taxonomic coverage during the last decades [see Huemer and Karsholt (1999, 2010) and bibliography in Huemer and Karsholt (2020)], some diverse genera, for example *Stomopteryx* Heinemann, 1870, *Aproaerema* Durrant, 1897, *Aristotelia* Hübner, 1825, and *Monochroa* Heinemann, 1870, still remain unrevised. In contrast, the genus *Caryocolum* has undergone extensive revisionary work with a constantly growing number of described species, currently 59 (Klimesch 1953–1954; Huemer 1988; Huemer and Karsholt 2010, 2020). However, after implementation of molecular data (DNA barcodes), Huemer et al. (2014) found clear indications of widespread, previously overlooked, cryptic diversity in the genus, documented for example in the recently revised *C.schleichi* species complex (Huemer 2020). In this paper *C.tricolorella* and allied species, a further case of underestimated alpha-diversity, are revised based on morphology and DNA barcodes, and two new species are described.

## ﻿Material and methods

The generic classification and the definition of species-groups follow Huemer (1988).

### ﻿Specimens

The study is based on about 140 specimens of the *C.tricolorella* subsection as part of the *C.interalbicella* species-group. Material was pinned and dried and either traditionally set or spread. Genitalia preparations followed standard techniques (Robinson 1976) adapted for the Gelechiidae as described by Pitkin (1986) and Huemer (1987).

Forewing length was measured from wing base to apex (including cilia) with an ocular micrometer, taking into account the smallest and largest specimen of available samples.

### ﻿DNA Barcodes

DNA barcode sequences are based on a 658 base-pair long segment of the mitochondrial COI gene (cytochrome c oxidase subunit 1). DNA samples (dried legs) were prepared according to the prescribed standards and successfully processed at the Canadian Centre for DNA Barcoding (**CCDB**, Biodiversity Institute of Ontario, University of Guelph) to obtain DNA barcodes using the standard high-throughput protocol described in deWaard et al. (2008). Altogether 106 successfully sequenced specimens of the *Caryocoluminteralbicella* species-group from BOLD (sequence length >600 bp, BIN available) are partially based on external sources (German Barcode of Life, Finnish Barcode of Life, Norwegian Barcode of Life, and others). These sequences cover 17 out of 18 species of the species-group, only leaving *Caryocolumnearcticum* without a DNA barcode. Twenty-seven sequences belong to the *Caryocolumtricolorella* species complex and details including complete voucher data and images of these specimens can be accessed in the public dataset “Lepidoptera of Europe – *Caryocolumtricolorella* species-group [DS-CARYTRIC]” in the Barcode of Life Data Systems BOLD (Ratnasingham and Hebert 2007). Sequences were finally submitted to GenBank.

Degrees of intra- and interspecific variation of DNA barcode fragments were calculated under the Kimura 2-parameter model of nucleotide substitution using analytical tools of BOLD Systems v. 4.0. (http://www.boldsystems.org). A neighbor-joining tree of DNA barcode data of central and south-eastern European taxa was constructed using MEGA6 (Tamura et al. 2013) under the Kimura 2-parameter model for nucleotide substitutions.

### ﻿Photographic documentation

Photographs of the adults were taken with an Olympus SZX 10 binocular microscope and an Olympus E 3 digital camera and developed using the software Helicon Focus v. 4.3 and Adobe Photoshop CS4 and Lightroom v. 2.3. Genitalia photographs were taken with an Olympus E1 Digital Camera through an Olympus BH2 microscope.

### ﻿Specimen repositories

**LMK** Landesmuseum Kärnten, Klagenfurt, Austria;

**NHM**Natural History Museum, London, United Kingdom;

**RCJL** Research collection Gerárd Labonne, Montpellier, France;

**RCJG** Research Collection Javier Gastón, Getxo, Spain;

**RCTM** Research Collection Toni Mayr, Feldkirch, Austria;

**TLMF**Tiroler Landesmuseum Ferdinandeum, Innsbruck, Austria;

**ZMUC** Zoological Museum, Natural History Museum of Denmark, Copenhagen, Denmark.

## ﻿Results

### ﻿Molecular analysis

**Figure 1.**
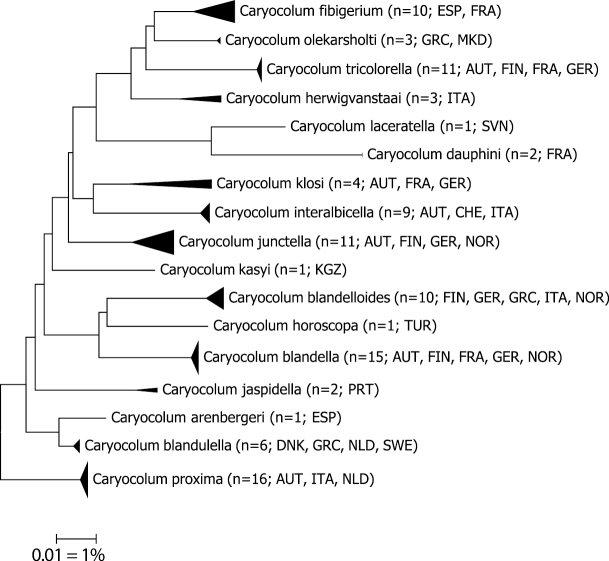
Neighbor-joining tree of species in the *Caryocoluminteralbicella* species group (Kimura 2-parameter, built with MEGA 6; Tamura et al. 2013), only sequences (>500 bp) considered. Note: the scale bar only applies to internal branches between species. Width of triangles represent sample size, depth the genetic variation within the cluster. Source: DNA Barcode data from BOLD (Barcode of Life Database; Ratnasingham 2018).

DNA sequencing resulted in a BIN concordant barcode fragment of >500 bp for 87 specimens and 17 species in the *Caryocoluminteralbicella* species group. Sequences of the COI barcode region revealed low intraspecific, but significantly higher interspecific, genetic distances (Table 1, Fig. 1). The normalized mean within-species divergence is 0.60% (SE 0.04). Only three species split in two BINs (Ratnasingham and Hebert 2013): *C.klosi*, *C.junctella*, and *C.herwigvanstaai* sp. nov., but it should be noted that the number of sequences is insufficient to estimate intraspecific variation for several species. A maximum intraspecific distance of 4.28% in *Caryocolumklosi* has to be re-assessed and may be due to unrecognized cryptic diversity. In contrast, minimum interspecific divergence is 1.55% in two BIN-sharing species but considerably higher in the remaining 15 species with a distance to the nearest neighbour ranging from 3.32% to 5.63%.

**Table 1.** Intraspecific mean K2P (Kimura 2-parameter) divergences, maximum pairwise distances, nearest species, nearest neighbour and distance to nearest neighbour (distances in %) in the *Caryocoluminteralbicella* species-group.

Species	Mean IntraSp	Max IntraSp	Nearest Species	Nearest Neighbour	Distance to NN
*Caryocolumarenbergeri*	N/A	0	*Caryocolumblandulella*	LEFIL287-10	1.55
*Caryocolumblandella*	0.12	0.36	*Caryocolumblandulella*	PHLAI019-12	5.04
*Caryocolumblandelloides*	0.25	0.98	*Caryocolumblandella*	GMGMM1305-14	5.29
*Caryocolumblandulella*	0.21	0.46	*Caryocolumarenbergeri*	LEASU109-18	1.55
*Caryocolumdauphini*	0	0	*Caryocolumlaceratella*	PHLAB900-10	5.63
*Caryocolumfibigerium*	0.89	2.41	*Caryocolumolekarsholti*	PHLAI014-12	3.37
*Caryocolumhoroscopa*	N/A	0	*Caryocolumblandella*	GMGMM1305-14	5.08
*Caryocoluminteralbicella*	0.34	0.77	*Caryocolumjunctella*	LEAST920-17	5.27
*Caryocolumjaspidella*	1.08	1.08	*Caryocolumblandulella*	PHLAI019-12	4.42
*Caryocolumjunctella*	1.12	2.34	*Caryocolumblandulella*	PHLAI019-12	4.03
*Caryocolumkasyi*	N/A	0	*Caryocolumjunctella*	LEAST920-17	4.91
*Caryocolumklosi*	2.16	4.28	*Caryocoluminteralbicella*	PHLAD577-11	5.43
*Caryocolumlaceratella*	N/A	0	*Caryocolumdauphini*	PHLAI447-13	5.63
*Caryocolumproxima*	0.36	1.08	*Caryocolumblandulella*	PHLAI019-12	3.8
*Caryocolumolekarsholti*	0.11	0.16	*Caryocolumfibigerium*	PHLAI403-13	3.37
*Caryocolumherwigvanstaai*	1.46	2.19	*Caryocolumolekarsholti*	PHLAI015-12	4.12
*Caryocolumtricolorella*	0.17	0.77	*Caryocolumolekarsholti*	PHLAI014-12	4.12

### ﻿Taxonomy

#### ﻿ Caryocolum

Taxon classificationAnimaliaLepidopteraGelechiidae

Gregor & Povolný, 1954

5AD432E7-49CE-5E3D-974E-83A20F24316F

Caryocolum Gregor and Povolný 1954: 8. Type species: Gelechialeucomelanella Zeller, 1839: 138.

##### *Caryocoluminteralbicella* species-group

The *Caryocoluminteralbicella* species-group was defined by Huemer (1988) and is characterized in the male genitalia by the following characters: uncus long and narrow; tegumen very broad anteriorly, strongly constricted medially, with large pedunculi; transtilla with spines; valva usually long and slender, subbasally strongly bent, apex frequently bulged, with brush of setae; sacculus knife-shaped; posterior margin of vinculum medially incised to broadly emarginated; saccus slender to moderately broad; phallus without cornuti. Female genitalia are characterized by the following characters: segment VIII with pair of ventral or dorsal processes, ventromedial area sclerotized with or without microtrichia; antrum short ring to long funnel; signum with a semi-oval basal plate and a strong distal hook. The species-group includes 18 species (Huemer 1988; Huemer and Karsholt 2010, 2020).

The informal *Caryocolumtricolorella* subsection is characterized by a long and evenly slender valva without apical bulge in the male genitalia, and a large, broadly funnel-shaped antrum in the female genitalia.

##### Checklist of *Caryoyoluminteralbicella* species-group

(species of the *C.tricolorella* species complex are marked with an asterisk; country of the type locality in brackets)

*Caryocolumklosi* (Rebel, 1917) (Austria)

*Caryocoluminteralbicella* (Herrich-Schäffer, 1854) (Switzerland)

*Caryocolumlaceratella* (Zeller, 1868) (Italy)

*Caryocolumdauphini* Grange & Nel, 2012 (France)

*Caryocolumnearcticum* Huemer, 1988 (USA)

*Caryocolumblandella* (Douglas, 1852) (UK, England)

*Caryocolumblandelloides* Karsholt, 1981 (Denmark)

*Caryocolumhoroscopa* (Meyrick, 1926) (India)

*Caryocolumjaspidella* (Chrétien, 1908) (Algeria)

*Caryocolumproxima* (Haworth, 1828) (UK, England)

*Caryocolumblandulella* (Tutt, 1887) (UK, England)

*Caryocolumarenbergeri* Huemer, 1989 (Spain)

*Caryocolumtricolorella* (Haworth, 1812)* (UK, England)

*Caryocolumfibigerium* Huemer, 1988* (Spain)

*Caryocolumherwigvanstaai* sp. nov.* (Italy)

*Caryocolumolekarsholti* sp. nov.* (Greece)

*Caryocolumjunctella* (Douglas, 1851) (UK, England)

*Caryocolumkasyi* Huemer, 1988 (Afghanistan)

#### ﻿ Caryocolum tricolorella

Taxon classificationAnimaliaLepidopteraGelechiidae

(Haworth, 1812)

509746E7-8E96-5E42-93D2-5A14B2705FE0

Tinea tricolorella Haworth 1812: 338. Syntypes, UK: England (NHM) [not traced].
Recurvaria contigua Haworth 1828: 552. Lectotype ♀, UK: England (NHM). Designated by Huemer (1988).
Gelechia acernella Herrich-Schäffer 1855: 185, pl. 77, fig. 580. Syntypes, Austria, Germany [not traced].

##### Other material.

[Austria] • 10 ♂; Burgenland, Jois 1.5 km NE; 200 m; 3 Aug 2021; [DNA barcode ids] TLMF Lep 30932, TLMF Lep 30933; P. Huemer leg.; • 1 ♂; Wien, Haschberg; 28 Jul 1915; all TLMF; [Germany] • 2 ♂, 1 ♀; Württemberg, Burgstall/Murr; 9–15 Jun 1973 e.l. (*Stellariaholostea*); L. Süssner leg; • 2 ♂; Württemberg, Kirchberg/Murr; 24 Jun 1963 e.l. (*Stellariaholostea*); [genitalia slide number] GU 86-032♂, P. Huemer; L. Süssner leg; • 2 ♂, 3 ♀; Württemberg, Markgröningen; 18–30 May 1961 e.l. (*Stellariaholostea*); L. Süssner leg; • 1 ♂; Württemberg, Markgröningen; 21 Jun 1963 e.l. (*Stellariaholostea*); L. Süssner leg; • 2 ♂; Württemberg, Markgröningen; 4–5 Jun 1964 e.l. (*Stellariaholostea*); L. Süssner leg; 2 ♂; Württemberg, Gronau, Kurzach Tal; 11–16 Jun 1973 e.l. (*Stellariaholostea*); [genitalia slide number] GEL 1092♀, P. Huemer; L. Süssner leg; 3 ♀; Württemberg, Schwarzwald, Sprollenmühle; 560 m; 18–22 Jun 1968 e.l. (*Stellariaholostea*); [genitalia slide number] GU 86-031♀, P. Huemer; L. Süssner leg; 1 ♂; Württemberg, Schwarzwald, Sprollenmühle; 550–580 m; 8 Jun 1967 e.l. (*Stellariaholostea*); L. Süssner leg; 1 ♂; Württemberg, Schwarzwald, Sprollenmühle; 560 m; 22 Jun 1969 e.l. (*Stellariaholostea*); L. Süssner leg; 5 ♂, 1 ♀; Württemberg, Schwarzwald, Bad Liebenzell; 450 m; 9–11 Jun 1971 e.l. (*Stellariaholostea*); [genitalia slide number] GEL 1288♂, P. Huemer; L. Süssner leg; all TLMF; [France] • 1 ♂; Midi-Pyrénées, Soulom; 31 Jul 2002; J. Nel leg.; TLMF; [Denmark] • 1 ♂, 2 ♀; Bótó; 22 Jul 1967; • 1 ♂; SZ, Vemmetofte; 9 May 1987 (larva) (*Stellariaholostea*); O. Karsholt leg.; all TLMF.

##### Diagnosis.

*Caryocolumtricolorella* differs from other species of the complex by its larger size and the extension of ochreous-orange scales on the dorsum and in the middle of the forewing. The male genitalia are characterized by the particularly long valva and sacculus, and the nearly straight posterior margin of the vinculum with indistinct lateromedial projections. The female genitalia differ from all other species by the distinctly smaller antrum.

##### Description.

**Figures 2–5.**
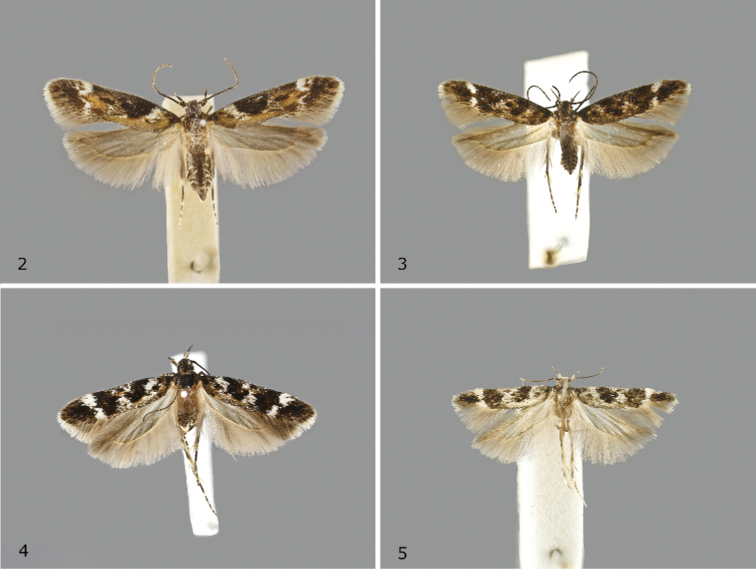
Adults **2***Caryocolumtricolorella*, male, Germany **3***C.fibigerium*, male, paratype, Spain **4***C.herwigvanstaai* sp. nov., male, holotype, Italy **5***C.olekarsholti*, male, holotype, Greece.

Adult (Fig. 2). Forewing length. ♂ 5.4–6.6 mm (ø = 5.92 mm, *n* = 5), ♀ 6.1–6.3 mm (ø = 6.20 mm, *n* = 5). Head with fuscous vertex, frons cream-white; second segment of labial palpus cream-white on inner and upper surface, predominantly grey-brown on outer surface, third segment dark brown with a few white scales particularly at apex; antenna black, weakly ringed whitish. Thorax and tegula dark brown anteriorly, posterior part intermixed ochreous. Abdomen dorsally grey, ventrally whitish, pale grey at margins. Forewing predominantly ochreous-orange with scattered white scales, costal and terminal area fuscous, distinct subtriangular black patch from fold to costa at about one-third and black dash distad of cell, dorsum ochreous-orange with concolorous extension towards costa at 1/5 and in middle at 3/4, inwardly lined with irregular white suffusion, larger white costal spot and smaller tornal dash separated by ochreous patch or by fuscous scales; cilia light grey with fuscous ciliary line, buff beyond line. Hindwing light grey, cilia greyish buff.

**Variation**: the wingspan varies from 10.0–14.5 mm [forewing length not stated] (Bland et al. 2002) showing a much greater variation than in the above examined material.

**Figures 6, 7.**
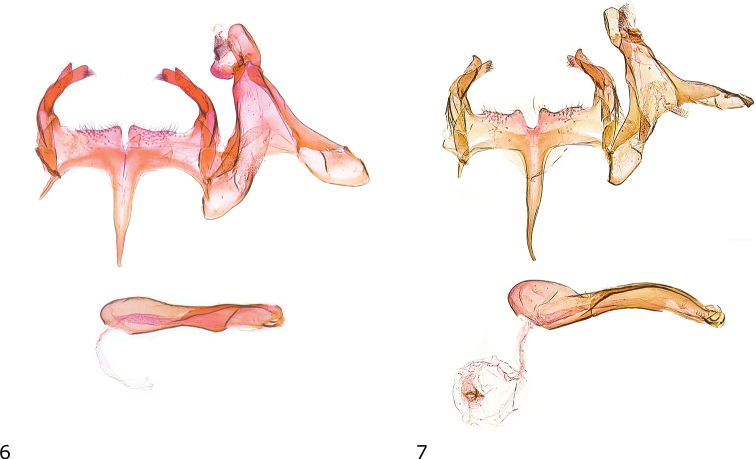
Male genitalia **6***Caryocolumtricolorella*, Germany, slide GEL 1218 P. Huemer **7***C.fibigerium*, Spain, slide GEL 1211 P. Huemer.

***Male genitalia*** (Fig. 6). Uncus long, suboval, posterior edges rounded; gnathos with large mesial sclerite, culcitula small; posterior third of tegumen slender, anterior part strongly widened towards broadly rounded pedunculi of about twice size of uncus, anterior margin with deep concave emargination; transtilla membranous with few microtrichia; valva basally curved ventrad, long, slender, apical part weakly broadened, apex with group of stiff setae; sacculus long, slightly shorter than valva but about same width, apex knife-shaped; vinculum wide and short, posterior margin moderately sclerotized, nearly straight, with shallow medial incision and hardly developed lateromedial projections, anterior margin with strongly sclerotized concave ridge; saccus slender, basally weakly widened, gradually narrowing towards pointed apex, about length of apex of valva to anterior margin of vinculum; anellus with pair of needle-shaped sclerites; phallus stout, almost straight, coecum weakly inflated, longitudinal ridge from about middle to apex, two small sclerotized hooklets at apex.

***Female genitalia*** (Fig. 10). Apophysis posterior about 4.5 times length of apophysis anterior; segment VIII smoothly sclerotized, with small dorsolateral flaps, posterior and inner edge strongly sclerotized, membranous ventromedial part with numerous microtrichia; apophysis anterior about three-quarters length of segment VIII; antrum comparatively short and small, about 4/5 length and 1/3 width of segment VIII between bases of apophyses anteriores, funnel-shaped; ductus bursae about twice length of apophysis anterior; corpus bursae semi-oval, signum with a large basal plate with long and slender hook.

##### Molecular data.

BIN: BOLD:AAF1506. The intraspecific average distance of the barcode region is 0.17%, the maximum distance 0.77% (*p*-distance) (*n* = 12). The minimum distance to the nearest neighbour, *C.olekarsholti* sp. nov., is 4.12%.

##### Distribution.

*Caryocolumtricolorella* is widely distributed from north-western Europe to Russia, extending to the central parts of the continent in the south, but most probably absent from the Mediterranean. All records from this area require verification and probably refer to other species.

##### Bionomics.

The biology of this species was described in detail by Stainton (1867), supplemented by several other authors (Sorhagen 1886; Schütze 1931; Hering 1935–1937). The young larva produces a gallery-like leaf-mine on *Stellariaholostea* or rarely on other *Stellaria* spp. (Caryophyllaceae), later feeding between spun shoots. *Cerastiumarvense* requires confirmation as another suspected hostplant. The larva has been observed from September to mid-April (Huemer 1988). Moths are on the wing from June to mid-September. The species prefers thermophilous forests and hedgerows at low elevation. This species is easily attracted to artificial light sources.

##### Remarks.

*Tineatricolorella* was described from an unspecified number of specimens from England (Haworth 1812) and is considered undisputed (Huemer 1988). The two junior synonyms are of taxa originating outside the geographic range of sibling species, namely *Recurvariacontigua* from England (Haworth 1828), and *Gelechiaacernella* described from Central Europe (Germany, Austria) and figured in detail in the original description (Herrich-Schäffer 1855).

#### ﻿ Caryocolum fibigerium

Taxon classificationAnimaliaLepidopteraGelechiidae

Huemer, 1988

F904404C-669E-52B4-B583-29EE225FA926

Caryocolum fibigerium Huemer 1988: 510, figs 86, 153, 214.

##### Type material.

***Holotype*.** [Spain] • ♀; Granada, Sierra Nevada, road to Veleta; 2200 m; 16 Jul 1962; K. Sattler leg; NHM.

***Paratypes*.** [Spain] • 2 ♂, 2 ♀; Andalucia, Sierra Nevada, Cam. d. Veleta; 2000 m; 24 Jul 1983; E. Traugott-Olsen leg.; • 9 ♂, 1 ♀; Andalucia, Sierra Nevada, Cam. d. Veleta; 2300 m; 19 Aug 1984; E. Traugott-Olsen leg.; all TLMF.

##### Other material.

[Spain] • 2 ♂; Andalucia, Sierra Nevada, Cam. d. Veleta; 2250 m; 1 Aug 1986; E. Traugott-Olsen leg.; • 1 ♂, 1 ♀; Andalucia, Sierra Nevada, Cam. d. Veleta; 2250 m; 3 Aug 1986; E. Traugott-Olsen leg.; • 1 ♂; Andalucia, Sierra Nevada, Cam. d. Veleta; 2250 m; 4 Aug 1986; E. Traugott-Olsen leg.; • 1 ♂; Andalucia, Sierra Nevada, Cam. d. Veleta; 2250 m; 4 Aug 1986; E. Traugott-Olsen leg.; • 2 ♂, 1 ♀; Andalucia, Sierra Nevada, Camino de la Veleta; 2250 m; 21 Jul 1985; [genitalia slide numbers] GEL 1211♂, GEL 1095♀, P. Huemer; G. Baldizzone and E. Traugott-Olsen leg.; • 1 ♂, 2 ♀; Castellon, Penygolosa N-Hang, Banyadera; 1500 m; 31 Aug 2005; [DNA barcode ids] BC TLMF Lep 03257, BC TLMF Lep 03258; P. Huemer leg.; • 4 ♂, 5 ♀; Alicante, Alcoj, Font Roja, W El Menejador, S-Hang; 1300 m; 4 Sep 2005; [DNA barcode ids] BC TLMF Lep 08899, BC TLMF Lep 08899; P. Huemer leg.; all TLMF; • 1 ♂; Almeria, Sierra de Gador; 2020 m; 31 Jul 2019; [genitalia slide number] 6810♂, J. Gastón, [DNA barcode id] TLMF Lep 30599; J. Gastón leg.; • 1 ♂, 1 ♀; Burgos, Castrobarto; 770 m; 13 Sep 2020; [genitalia slide numbers] 8273♂, J. Gastón, 8253♀, J. Gastón [DNA barcode ids] TLMF Lep 30600, TLMF Lep 30601; J. Gastón leg.; all RCJG; [France] • 1 ♂; Languedoc-Rousillon, Dourbies, Lac de Pises; 1300 m; 13 Sep 2020; [genitalia slide number] Gla 020/1984♂, G. Labonne, [DNA barcode id] TLMF Lep 30991; G. Labonne leg.; 1 ♀; Languedoc-Rousillon, Le Caylar; 740 m; 25 Aug 2016; [genitalia slide number] Gla 016/2825♀, G. Labonne, [DNA barcode id] TLMF Lep 30990; G. Labonne leg.; all RCGL; • 1 ♂; Hautes Pyrénées, Pic du Midi de Bigorre; 2400 m; 7 Aug 2002; [genitalia slide number] 14427♂, J. Nel; [DNA barcode id] BC TLMF Lep 06904; J. Nel leg.; • 1 ♂; Cantal, Lessenat; 700 m; 10 Aug 1995; [genitalia slide number] 3610♂, J. Nel; J. Nel. leg.; • 1 ♂; Alpes Maritimes, Caussols; 1100 m; 14 Aug 1971; [genitalia slide number] GU 88/136♂, P. Huemer; F. Dujardin leg; 1 ♂; Alpes Maritimes, Col de Vence; 11–12 Jun 1981; 1100 m; F. Hahn leg; • 1 ♂; Basses-Alpes, Montagne de Lure; 1500 m; 20 Jul 1992; J. Nel leg.; • 1 ♂; Basses-Alpes, Montagne de Lure; 1720 m; 8 Jun 1994 e.l. (*Cerastium*); [genitalia slide number] 2035♂, J. Nel; J. Nel leg.; • 1 ♂, 1 ♀; Var, Rougiers, Val. de Pourien; 28 Apr 1994 e.l. (*Cerastium*); [genitalia slide numbers] 1944♂, 1945♀, J. Nel; J. Nel leg.; all TLMF.

##### Diagnosis.

*Caryocolumfibigerium* differs from *C.tricolorella* by its distinctly smaller size on average and the less extensive ochreous markings. It can be distinguished from *C.herwigvanstaai* and *C.olekarsholti* by the smaller, white costal and tornal spots and the reduced white mottling of the medial and subbasal fasciae. The male genitalia differ from *C.tricolorella* in the shorter valva and sacculus and the additional humps of the posterior margin of the vinculum. *Caryocolumfibigerium* is very similar to *C.herwigvanstaai* and *C.olekarsholti* in this character, but with a weakly developed lateral hump. Furthermore, the sacculus is wider than in *C.herwigvanstaai*. The antrum of the female genitalia is much larger than in *C.tricolorella* and also in the latter two species, exceeding the length of the apophysis anterior, furthermore the dorsolateral flaps of segment VIII are larger compared to *C.herwigvanstaai* and *C.olekarsholti*.

##### Description.

Adult (Fig. 3). Forewing length. ♂ 4.8–6.2 mm (ø = 5.30 mm, *n* = 5), ♀ 4.6–5.1 mm (ø = 4.90 mm, *n* = 5). Head with fuscous vertex, frons cream-white; second segment of labial palpus cream-white on inner and upper surface, predominantly grey-brown on outer surface, third segment dark brown with a few white scales particularly at apex; antenna black, weakly ringed whitish. Thorax and tegula dark brown occasionally slightly intermixed ochreous. Abdomen dorsally grey, ventrally whitish, pale grey at margins. Forewing predominantly fuscous in costal and terminal area, dorsum mixed fuscous and ochreous with scattered white scales, extending into middle of wing particularly at 1/5 and at about middle of wing, distinct white costal and tornal spots separated by ochreous or fuscous scales, irregularly shaped black patch from fold to costa at about 1/3 interrupted by ochreous scales, black plical and discal spot; cilia light grey with fuscous ciliary line, buff beyond line. Hindwing light grey, cilia greyish buff.

**Variation**: the extent of ochreous scales varies considerably and occasionally they are completely absent. Specimens from the Hautes Pyrénées and Alps are larger on average than those from southern Spain with fewer ochreous scales.

***Male genitalia*** (Fig. 7). Uncus long, suboval, posterior edges rounded; gnathos with large mesial sclerite, culcitula small; posterior 1/3 of tegumen slender, anterior part strongly widened towards broadly rounded pedunculi of about twice size of uncus, anterior margin with deep concave emargination; transtilla membranous with few microtrichia; valva basally curved ventrad, moderately short, slender, apical part weakly constricted, oblique apex with group of stiff setae; sacculus long, nearly length and width of valva, apex rounded, with dorsally pointed projection; vinculum wide and short, posterior margin moderately sclerotized, with shallow medial incision and distinctly rounded lateromedial projections, lateral projections shallow, anterior margin with strongly sclerotized concave ridge; saccus slender, basally weakly widened, gradually narrowing towards pointed apex, slightly exceeding length of apex of valva to anterior margin of vinculum; anellus with pair of needle-shaped sclerites; phallus stout, distal part weakly curved and contorted, coecum weakly inflated, longitudinal ridge from about middle to apex, two small sclerotized hooklets at apex.

***Female genitalia*** (Fig. 11). Apophysis posterior about 4 times length of apophysis anterior; segment VIII with suboval sclerotized dorsolateral zones, with distinct dorsolateral flaps, posterior and inner edge strongly sclerotized, membranous ventromedial part with numerous microtrichia; apophysis anterior about length of segment VIII; antrum large, funnel-shaped, slightly extending beyond apex of apophysis anterior and basally 2/3 width of segment VIII between bases of apophyses anteriores, posterior edge weakly convex; ductus bursae about twice length of apophysis anterior; corpus bursae semi-oval, signum a crescent-shaped basal plate with moderately long and stout hook.

##### Molecular data.

BIN: BOLD:AAU3076. A genetically variable species, mainly due to a deviating specimen from Spain. The intraspecific average distance of the barcode region is 0.89%, the maximum distance 2.41% (*p*-distance) (*n* = 11) with all sequences clustering in a single BIN. The minimum distance to the nearest neighbour, *C.olekarsholti* sp. nov., is 3.37%.

##### Distribution.

*Caryocolumfibigerium* in its current taxonomic sense is confirmed from the Iberian Peninsula (Spain) and southern parts of France (Huemer and Karsholt 2010), whereas other published records from Morocco (Huemer 1988), Portugal (Corley 2015), and northern Italy (Karsholt and Huemer 1995) require re-examination including DNA barcode analysis.

##### Bionomics.

In Portugal the larva has been found from November to mid-December on *Arenariamontana*, living between two spun leaves, usually at tip of a shoot. Young larvae are suspected as probable leaf-miners (Corley 2002). However, identity of these populations has to be re-assessed. Unpublished breedings from France from *Cerastium* sp. by Jacques Nel show a possibly wider spectrum of host-plants. The adults have been found in from early June to early September at artificial light sources near rock and scree at altitudes of about 700–2400 m.

##### Remarks.

*Caryocolumfibigerium* was described from two disjunct Mediterranean areas, from Morocco to Spain and from Bulgaria to Greece, with the holotype from southern Spain. However, this study indicates that material from Morocco requires verification, populations from the Balkans belong to *C.olekarsholti*, and unpublished records from central Italy are *C.herwigvanstaai*.

#### ﻿ Caryocolum herwigvanstaai sp. nov.

Taxon classificationAnimaliaLepidopteraGelechiidae

58E3D93A-EB8A-56F3-8AC1-8292CCD29472

http://zoobank.org/5C8F64C1-7008-4356-8CAC-A775D3F05D12

##### Type material.

***Holotype*.** [Italy] • ♂; L’Aquila, NP Gran Sasso, ex Miniera di Lignite; 1750 m; 14–15 Jul 2010; [genitalia slide number] GEL 1153♂, P. Huemer; P. Huemer leg; TLMF.

***Paratypes*.** [Italy] • 5 ♂, 5 ♀; same collection data as for holotype; [genitalia slide number] GEL 1155♀, P. Huemer; [1 ♂, 1 ♀ genitalia in glycerin capsule]; [DNA barcode ids] BC TLMF Lep 01600; all TLMF; • 10 ♂, 3 ♀; same collection data as for holotype; 1750 m; 15 Jul 2010; T. Mayr leg.; RCTM; • 1 ♂; same collection data as for holotype; 1750 m; 14 Jul 2010; T. Mayr leg.; RCTM; • 1 ♀; Rieti, Monte Terminillo; 1730–1780 m; 11 Jul 2010; P. Huemer leg.; [DNA barcode ids] BC TLMF Lep 01601; • 6 ♂; Rieti, Monte Terminillo; 1700 m; 17 Jul 2011; T. Mayr leg.; RCTM; • 1 ♀; Chieti, PN della Majella, Taranta Peligna, Pian di Valle; 770 m; 20 Jul 2011; P. Huemer leg.; BC TLMF Lep 05038; all TLMF.

##### Diagnosis.

*Caryocolumherwigvanstaai* differs from *C.tricolorella* by its distinctly smaller size and the less extensive ochreous-orange markings, and from *C.fibigerium* by the extended white forewing markings which are, however, less pronounced at the inner margin compared to *C.olekarsholti*. The male genitalia differ from *C.tricolorella* by the shorter valva and sacculus and the additional, although moderately low, humps of the posterior margin of the vinculum. From *C.fibigeriumC.herwigvanstaai* differs in particular by the more slender sacculus and the distinct lateral humps of the posterior margin of the vinculum, and from *C.olekarsholti* by the apically slightly dilated valva and the slender sacculus. The antrum of the female genitalia is much larger than in *C.tricolorella* but smaller than in *C.fibigerium*, not extending the length of apophysis anterior. The anterior margin of the antrum is concave in *C.herwigvanstaai* but convex in *C.olekarsholti*.

##### Description.

Adult (Fig. 4). Forewing length. ♂ 4.9–5.5 mm (ø = 5.25 mm, *n* = 4), ♀ 5.1–5.7 mm (ø = 5.40 mm, *n* = 4). Head with fuscous vertex, frons cream-white; second segment of labial palpus cream-white on inner and upper surface, predominantly grey-brown on outer surface, third segment dark brown with a few white scales particularly at apex; antenna black, weakly ringed whitish. Thorax and tegula dark brown with a few intermixed ochreous scales. Abdomen dorsally grey, ventrally whitish, pale grey at margins. Forewing predominantly fuscous in costal and terminal area, dorsum mixed ochreous-whitish with scattered fuscous scales, extensive white mottling from dorsum to costa at 1/5 and 1/2, large white costal and tornal spots nearly fused, separated by few fuscous scales, irregularly shaped black patch from fold to costa at about 1/3 interrupted by ochreous scales, black plical and discal spot; cilia light grey with fuscous ciliary line, buff-whitish beyond line. Hindwing light grey, cilia greyish buff.

**Variation**: the extent of ochreous scales, particularly along the dorsum, is slightly variable.

**Figures 8, 9.**
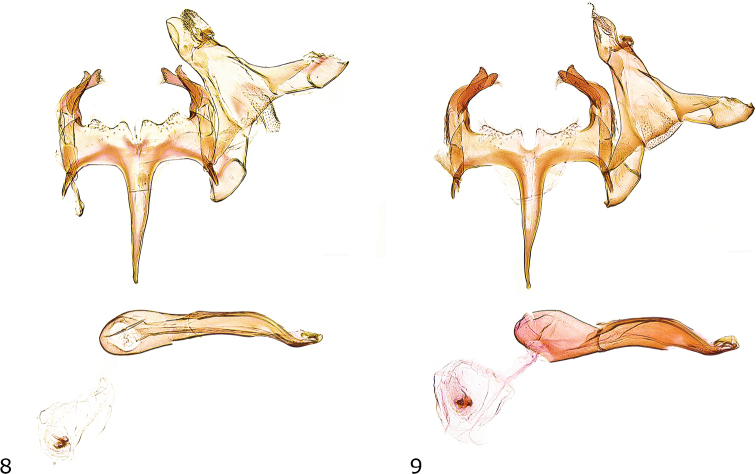
Male genitalia **8***C.herwigvanstaai* sp. nov., holotype, Italy, slide GEL 1153 P. Huemer **9***C.olekarsholti*, paratype, Greece, slide GEL 1213 P. Huemer;

***Male genitalia*** (Fig. 8). Uncus long, suboval, posterior edges rounded; gnathos with large mesial sclerite, culcitula small; posterior 1/3 of tegumen slender, anterior part strongly widened towards broadly rounded pedunculi of about twice size of uncus, anterior margin with deep concave emargination; transtilla membranous with few microtrichia; valva basally curved ventrad, moderately short, slender, apical part slightly dilated, obliquely pointed apex with group of stiff setae; sacculus moderately long, more slender and shorter than valva, apex rounded, with dorsally pointed projection; vinculum wide and short, posterior margin moderately sclerotized, with shallow medial incision and distinctly rounded lateromedial projections, lateral projections distinct, anterior margin with strongly sclerotized concave ridge; saccus slender, basally weakly widened, gradually narrowing towards pointed apex, slightly exceeding length of apex of valva to anterior margin of vinculum; anellus with pair of needle-shaped sclerites; phallus stout, distal part weakly curved and contorted, coecum weakly inflated, longitudinal ridge from about middle to apex, two small sclerotized hooklets at apex.

**Figures 10, 11.**
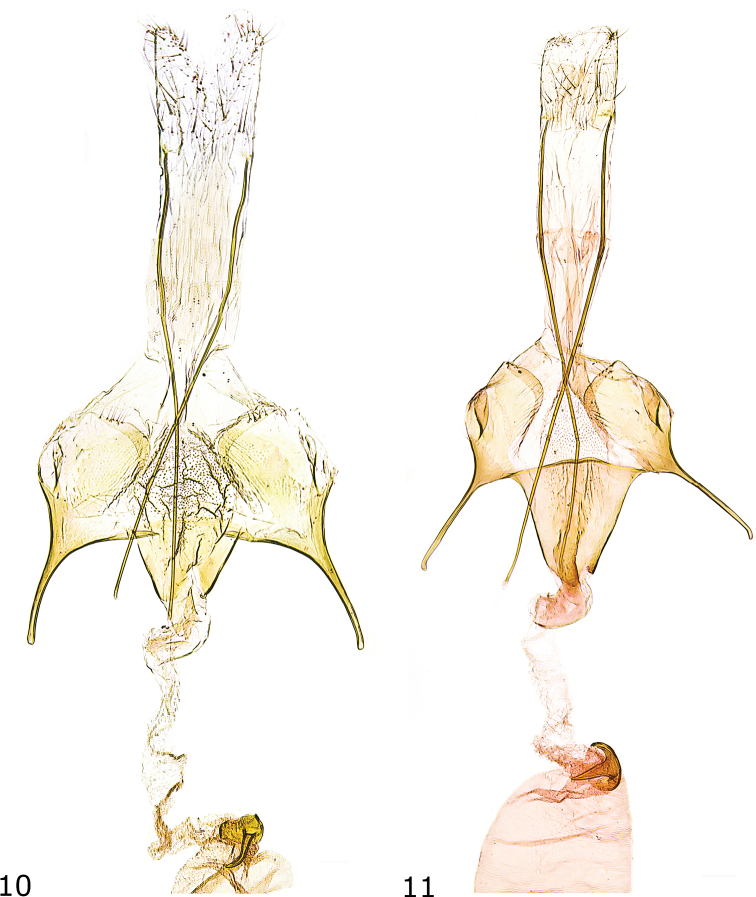
Female genitalia **10***Caryocolumtricolorella*, Germany, slide GEL 1092 P. Huemer **11***C.fibigerium*, Spain, slide GEL 1095 P. Huemer.

**Figures 12, 13.**
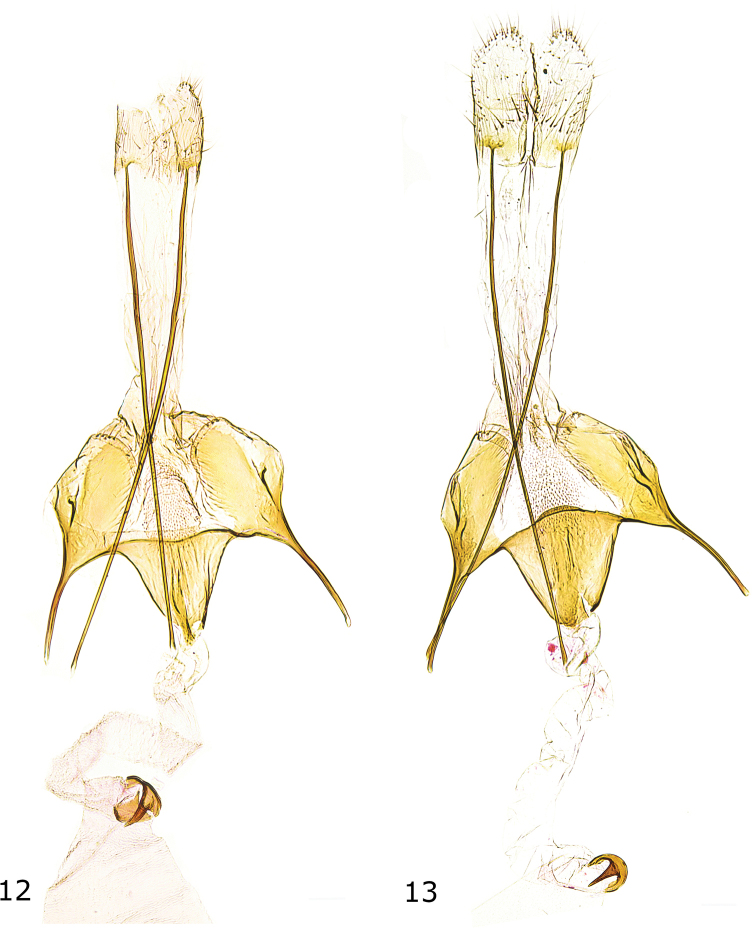
Female genitalia **12***C.herwigvanstaai* sp. nov., paratype, Italy, slide GEL 1155 P. Huemer **13***C.olekarsholti*, paratype, Greece, slide GEL 1231 P. Huemer.

***Female genitalia*** (Fig. 12). Apophysis posterior about 4.5 times length of apophysis anterior; segment VIII with suboval sclerotized dorsolateral zones, with small dorsolateral flaps, posterior and inner edge strongly sclerotized, membranous ventromedial part with numerous microtrichia; apophysis anterior about length of segment VIII; antrum moderately large, funnel-shaped, shorter than apophysis anterior and segment VIII, basally about 1/2 width of segment VIII between bases of apophyses anteriores, posterior edge weakly concave; ductus bursae about twice length of apophysis anterior; corpus bursae semi-oval, signum a crescent-shaped basal plate with moderately long and stout hook.

##### Molecular data.

BINs: BOLD:AAO2674, BOLD:ADK9243. A genetically variable species splitting into two BINs which, however, require re-evaluation from additional material. The distance between both BINs is 2.1% (*n* = 3). The minimum distance to the nearest neighbour, *C.olekarsholti*, is 4.12%.

##### Etymology.

The species is dedicated to DDr Herwig van Staa (Innsbruck, Austria), former governor of the province of Tyrol on his 80^th^ birthday on the 10 June 2022, and in recognition of his tremendous support of the Tyrolean Federal State Museums and the Alpenzoo Innsbruck, resulting in a joint Natural History Museum.

##### Distribution.

The species is currently only known from Central Italy but may have a wider distribution on the Italian Peninsula. Mariani (1943) had published a record of *C.tricolorella* from Sicily, which possibly is *C.herwigvanstaai*.

##### Bionomics.

Host-plant and early stages are undescribed but it seems most likely that the species shows a similar behaviour as related taxa with the potential host-plant among *Cerastium* or related genera of Caryophyllaceae. The adults have been found in mid-July at artificial light sources near rock and scree on calcareous soil at altitudes of about 1700–1800 m.

#### ﻿ Caryocolum olekarsholti sp. nov.

Taxon classificationAnimaliaLepidopteraGelechiidae

E6CF7E40-DCA8-595B-9F3F-7FC46DD3BB90

http://zoobank.org/52FA17A1-02D7-41BD-8B5B-A6BA384D3E4A

##### Type material.

***Holotype*.** [Greece] • ♂; Ioannina, Psorovouni NE, Vradheto; 1750 m; 4 Aug 2012; [genitalia slide number] GEL 1209♂, P. Huemer; C. Wieser leg; LMK.

***Paratypes*.** [Greece] • 18 ♂, 11 ♀; same collection data as for holotype; [genitalia slide numbers] GEL 1213♂, GEL 1233♀, P. Huemer; [DNA barcode ids] KLM Lep 00489, KLM Lep 00490, BC TLMF Lep 05038; all KLM; • 1 ♂; Trikala, Katara pass; 1700 m; 13 Jul 1998; [genitalia in glycerin capsule]; M. Egger leg.; TLMF; 4 ♂; Ioannina, Katar pass; 1600 m; 11 Aug 1985; M. Fibiger leg.; all ZMUC; [North Macedonia] • 1 ♂, 2 ♀; Tetovo, Popova Sapka, W Tetovo; 2130 m; 7 Aug 2012; [DNA barcode ids] KLM Lep 00488; C. Wieser leg.; all KLM; [Bulgaria] • 1 ♂; Samokov; 4 Jul 1911; [unknown collector]; NHM.

##### Diagnosis.

*Caryocolumolekarsholti* differs from *C.tricolorella* by its distinctly smaller size and the lack of ochreous-orange markings, and from the other species of the complex by the pronounced white forewing markings with few or completely absent ochreous scales. The male genitalia differ from *C.tricolorella* by the shorter valva and sacculus and the additional humps of the posterior margin of the vinculum. *Caryocolumolekarsholti* is very similar to *C.fibigerium*, with only subtle diagnostic characters such as the more distinct lateral projection of the posterior margin of the vinculum and the distally weakly dilated sacculus. *Caryocolumolekarsholti* differs from *C.herwigvanstaai* in particular by the distinctly broader sacculus and the distally almost parallel-sided valva. The antrum of the female genitalia is much larger in *C.olekarsholti* than in *C.tricolorella* but smaller than in *C.fibigerium*, not extending the length of the apophysis anterior. The anterior margin of the antrum is convex in *C.olekarsholti* but concave in *C.herwigvanstaai*.

##### Description.

Adult (Fig. 5). Forewing length. ♂ 4.7–4.9 mm (ø = 4.83 mm, *n* = 4), ♀ 4.7–4.8 mm (ø = 4.73 mm, *n* = 4). Head with fuscous vertex, frons cream-white; second segment of labial palpus cream-white on inner and upper surface, predominantly grey-brown on outer surface, third segment dark brown with a few white scales particularly at apex; antenna black, weakly ringed whitish. Thorax and tegula dark brown, intermixed with light grey. Abdomen dorsally grey, ventrally whitish, pale grey at margins. Forewing predominantly fuscous in costal and terminal area, ochreous scales absent or largely reduced, dorsum whitish with scattered fuscous scales, extensive white mottling from dorsum to costa at 1/5 and 1/2, large white costal and tornal spots nearly fused, separated by a few fuscous scales, irregularly shaped black patch from fold to costa at about 1/3, indistinct black plical and discal spots; cilia light grey with fuscous ciliary line, buff-whitish beyond line. Hindwing light grey, cilia greyish buff.

**Variation**: the extent of white scales, particularly along dorsum, varies considerably.

***Male genitalia*** (Fig. 9). Uncus long, suboval, posterior edges rounded; gnathos with large mesial sclerite, culcitula small; posterior third of tegumen slender, anterior part strongly widened towards broadly rounded pedunculi of about twice size of uncus, anterior margin with deep concave emargination; transtilla membranous with few microtrichia; valva basally curved ventrad, moderately short, slender, apical part weakly constricted, oblique apex with group of stiff setae; sacculus long, nearly length and width of valva, distally weakly dilated, apex rounded, with dorsally pointed projection; vinculum wide and short, posterior margin moderately sclerotized, with shallow medial incision and distinctly rounded lateromedial and lateral projections, anterior margin with strongly sclerotized concave ridge; saccus slender, basally weakly widened, gradually narrowed towards pointed apex, slightly exceeding length of apex of valva to anterior margin of vinculum; anellus with pair of needle-shaped sclerites; phallus stout, distal part weakly curved and contorted, coecum weakly inflated, longitudinal ridge from about middle to apex, two small sclerotized hooklets at apex.

***Female genitalia*** (Fig. 13). Apophysis posterior about 5 times length of apophysis anterior; segment VIII with suboval sclerotized dorsolateral zones, with distinct dorsolateral flaps, posterior and inner edge strongly sclerotized, membranous ventromedial part with numerous microtrichia; apophysis anterior about length of segment VIII; antrum moderately large, funnel-shaped, shorter than apophysis anterior and segment VIII, about 1/2 width of segment VIII between bases of apophyses anteriores, posterior edge convex; ductus bursae about twice length of apophysis anterior; corpus bursae semi-oval, signum a crescent-shaped basal plate with moderately long and stout hook.

##### Molecular data.

BIN: BOLD:ACC2659. The intraspecific average distance of the barcode region is 0.11%, the maximum distance 0.16% (*p*-distance) (*n* = 3). The minimum distance to the nearest neighbour, *C.fibigerium*, is 3.37%.

##### Etymology.

The species is named in honour of Ole Karsholt (Copenhagen, Denmark) in recognition of his outstanding contribution to the systematics and taxonomy of European Gelechiidae.

##### Distribution.

The species is currently only known from Bulgaria, Greece, and North Macedonia but is probably more widely distributed on the Balkan Peninsula.

##### Bionomics.

Host-plant and early stages are undescribed, but it seems most likely that the species shows a similar behaviour as related taxa with the potential host-plant among *Cerastium* and/or *Stellaria* spp. The adults have been found from mid-July to early August at artificial light sources in mountainous habitats dominated by rock and scree on calcareous soil.

## ﻿Discussion

Cryptic diversity has been found in many different families of European Lepidoptera during the last years, progress mainly driven by the implementation of molecular methods and newly collected samples resulting from better access to remote parts of the continent. The majority of cryptic species seems to be hidden among various groups of so-called traditional “micromoths” (Huemer et al. 2020; Lopez-Vaamonde et al. 2021), whereas only a few overlooked species have been detected in the more “spectacular” taxonomic groups such as Papilionoidea (Dincă et al. 2021) or recently in the “macromoths” (Ronkay and Huemer 2018; Šumpich and Jagelka 2021). The majority of newly detected cryptic species seems to occur in allopatry, particularly in mountain areas of southern Europe, and they often cause ongoing taxonomic problems (Mutanen et al. 2012). In contrast only moderately few sibling species have been found in sympatry (Hernández-Roldán et al. 2016; Mutanen et al. 2020; Berggren et al. 2022).

The likely reasons for increased diversification in the southern part of the continent date back to the Messinian crisis approximately 5.96–5.33 mya and the consequent reflooding of the Mediterranean Sea with the establishment of a Mediterranean climate (Hewitt 2011; Fiz-Palacios and Valcárcel 2013; Carnicero et al. 2017). Furthermore, Pleistocene glaciation processes, which began about 2.5 mya, led to increased isolation of fragmented landscapes with temporary connections and disconnections and thus favouring speciation processes (Médail and Diadema 2009; Morales-Barbero et al. 2018). Vicariant distribution patterns of closely related Lepidoptera in southern Europe may reflect classical Pleistocene macrorefugia for European temperate species in the Iberian, Italian, and Balkan Peninsulas. The current distribution of the *C.tricolorella* species-complex with three species restricted to the three major Mediterranean peninsulas perfectly matches this scenario. However, the taxonomic complexity had not been recognized until now and only two species were formerly separated, with *C.fibigerium* considered as a Holomediterranean and *C.tricolorella* as a Central and Northern European species (Huemer 1988). Unexpectedly, re-assessment of molecular and morphological traits supported the existence of four as opposed to two species. In particular DNA barcodes have been of essential value in resolving the taxonomy of this species complex which is supported by rather subtle morphological characters. Similarly, several cryptic species of *Caryocolum* have been recently detected (Huemer et al. 2014; Huemer 2020). These studies had already indicated that revisionary work was still required on additional species (*C.peregrinella* and *C.klosi*) of this diverse genus with an exceptionally large intraspecific barcode divergence.

## Supplementary Material

XML Treatment for Caryocolum

XML Treatment for Caryocolum tricolorella

XML Treatment for Caryocolum fibigerium

XML Treatment for Caryocolum herwigvanstaai

XML Treatment for Caryocolum olekarsholti
